# Feature–Classifier Pairing Compatibility for sEMG Signals in Hand Gesture Recognition under Joint Effects of Processing Procedures

**DOI:** 10.3390/bioengineering9110634

**Published:** 2022-11-02

**Authors:** Mohammed Asfour, Carlo Menon, Xianta Jiang

**Affiliations:** 1 Ubiquitous Computing and Machine Learning Lab, Department of Computer Science, Memorial University of Newfoundland and Labrador, St. John’s, NL A1C 5S7, Canada; 2 Biomedical and Mobile Health Technology Laboratory, Department of Health Sciences and Technology, ETH Zürich, 8008 Zürich, Switzerland; 3 Menrva Research Group, Schools of Mechatronic Systems Engineering and Engineering Science, Simon Fraser University, Vancouver, BC V6B 5K3, Canada

**Keywords:** analysis, classification methods, electromyography, hand gestures, signal processing

## Abstract

Gesture recognition using surface electromyography (sEMG) serves many applications, from human–machine interfaces to prosthesis control. Many features have been adopted to enhance recognition accuracy. However, studies mostly compare features under a prechosen feature window size or a classifier, biased to a specific application. The bias is evident in the reported accuracy drop, around 10%, from offline gesture recognition in experiment settings to real-time clinical environment studies. This paper explores the feature–classifier pairing compatibility for sEMG. We demonstrate that it is the primary determinant of gesture recognition accuracy under various window sizes and normalization ranges, thus removing application bias. The proposed pairing ranking provides a guideline for choosing the proper feature or classifier in future research. For instance, random forest (RF) performed best, with a mean accuracy of around 74.0%; however, it was optimal with the mean absolute value feature (MAV), giving 86.8% accuracy. Additionally, our ranking showed that the proper pairing enables low-computational models to surpass complex ones. The Histogram feature with linear discriminant analysis classifier (HIST-LDA) was the top pair with 88.6% accuracy. We also concluded that a 1250 ms window and a (−1, 1) signal normalization were the optimal procedures for gesture recognition on the used dataset.

## 1. Introduction

Hand gesture classification is an essential means of interaction and control for humans across many fields such as virtual reality (VR) [1], control of robotic arms [2], or hand prostheses [3]. Many methods are used to detect hand gestures, such as visual methods using cameras [4], kinematic methods using sensors activated by motion [5], brain electrical activity [6], and muscles signal activation methods utilizing surface electromyography sensors (sEMG) [7,8]. Among these methods, sEMG has excellent advantages and has been prominently used in medical devices, human–machine interaction [2,9], and prosthesis control [10,11], as it provides a safe, easy-to-use, and noninvasive way to measure the energy of muscles.

The relatively new progress in several fields made electromyography sensors more affordable and sensitive. For instance, Prakash et al. [12] developed an sEMG armband that obtained a 1.4-times greater signal-to-noise ratio (SNR) on average and a 45% increase in sensitivity compared to a commercially available EMG sensor. Thanks to those advancements, sEMG has become a more reliant gesture recognition option for various applications. Dwivedi et al. [1] proposed a virtual reality experience to rehabilitate upper limb amputees via sEMG hand gesture classification with a random forest classifier. They obtained a representation of the manipulated object movement in VR, achieving up to 92% accuracy while performing the gestures, reaching up to 83% classification accuracy, after optimizing the feature window size, its stride, and the number of base models of the classifier.

More studies explored other aspects of sEMG gesture recognition. Khushaba et al. [13] investigated the aspect of the window size and its effect with a varying number of EMG channels. They examined window sizes varying from 32 ms to 256 ms and the number of channels varying from 8 to 128/256. They concluded that high-definition EMG setups with numerous channels could obtain good performance with small window sizes. In another study, Chen et al. [14] investigated multiple models with varying window sizes, showing that some models outperform others when the window size is expanded.

Due to dependency on sEMG signals in such applications [15], their classification and processing procedures were extensively investigated [16,17], such as features [18], using very common features for sEMG. Nevertheless, the bulk of the literature focuses on a prespecified control environment, which limits the generalization of the findings outside the chosen settings, such as the classifier used. For instance, Phinyomark et al. [19] investigated the effects of different sEMG features, such as mean absolute value (MAV) and waveform length (WL). They found some features redundant while recommending others. However, they analyzed all time domain features using the linear discriminant analysis classifier (LDA) only under a fixed feature window size, not including the classifier’s complexity or bias effect.

From an application perspective, most studies consider a prechosen application, rendering their results incompatible with other ones. For example, offline gesture recognition studies, with large windows, usually obtain around 95% accuracy [20,21], while real-time studies, with small windows, obtain around 80–85% accuracy [22]. Benalcazar et al. [23] proposed a multi-stage model of signal acquisition, preprocessing, features, classification, and postprocessing. Using the KNN classifier, they surpassed a commercial armband’s accuracy by 3% on five gestures. They used a 1 s window with a 250 ms stride, as they agreed that real-time classification has to be under 300 ms.

Hence, the distinct settings in the literature raise the need for a comprehensive study of the variations in sEMG processing concurrently with the fewest predetermined conditions. A thorough analysis would encompass the feature choice and the complexity of the classification method effects on accuracy while simultaneously exploring the feature window size and preprocessing steps such as the signal normalization range. Such an analysis would serve as a solid base for other research points and applications regardless of their study settings.

In this regard, Mendes Junior et al. [24] provided a broad study, investigating multiple classifiers using sEMG features from the literature. They used a feature selection technique to determine the optimally combined features for each classifier. Moreover, they illustrated the impact of multiple dimensionality reduction techniques on classification as a preprocessing step. However, without a standard window size, their techniques, using a 2000 ms window, can be computationally expensive and cause perceptible delay for real-time gesture classification. This limitation was shown by Smith et al. [25], who confirmed an inversely proportional relationship between classification error and the window size. Thus, their results are not generalizable to other window sizes.

This study proposes the existence of feature–classifier pairing compatibility, which radically governs the recognition performance and was tested with varying window sizes and normalization ranges to obtain the best settings for various research points and applications without bias. Secondly, we suggest that the window size is optimal for gesture recognition in a range beyond the window sizes used in real-time applications. As a final investigation point, we expected a normalization range that maintains signal polarity, including more information, to be optimal for most feature–model pairs. From several normalization formulas [26,27,28], we used the task peak values, as they suit the scope of gesture recognition.

By investigating the pairing compatibility in different scenarios, we removed any bias towards a prechosen application or circumstance; thus, the results become generalizable. We discuss the findings of the combinatorial settings and rank them by performance. Consequently, we deduce from the paper’s hypotheses that a compatible feature–model pair with a moderately large window will surpass any configuration. Research that benefits from this analysis varies from real-time with a small window to offline recognition and from the limited computation using linear models to higher-end systems with ensemble models without much delay.

## 2. Materials and Methods

### 2.1. Data Collection

**Figure 1:**
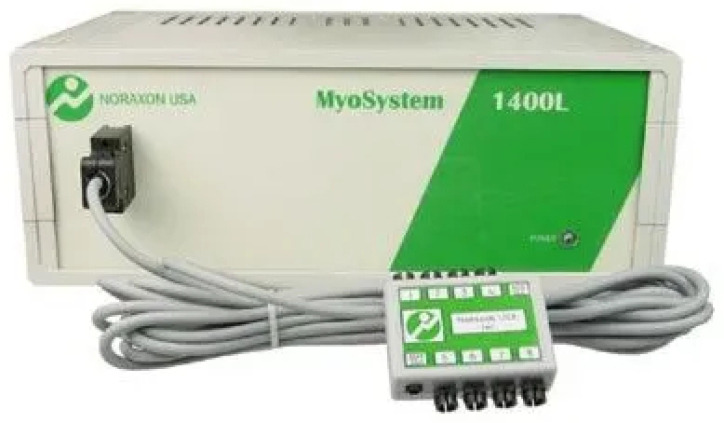
The MyoSystem 1400L sEMG data acquisition device.

**Figure 2:**
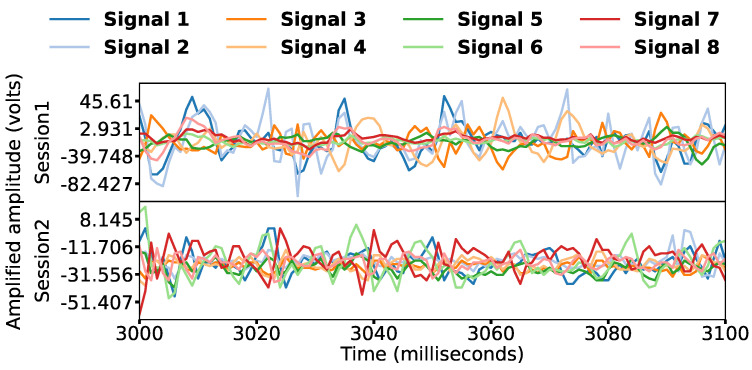
Samples of Participant 4’s acquired sEMG signals.

**Figure 3:**
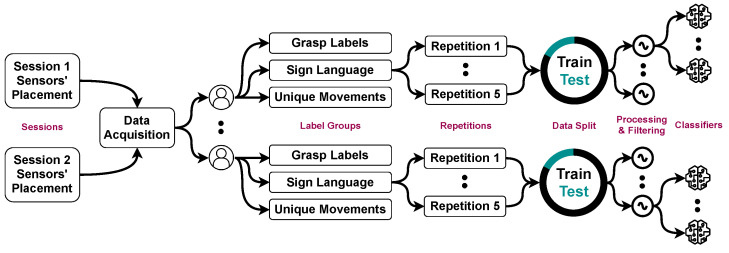
A diagram showing the breakdown of the data categories in the dataset.

The data were from a previous study [29] using an acquisition device from Noraxon (Scottsdale, AZ, USA) (Myosystem 1400 L) in Figure 1 from 12 fully operational right-handed participants, six males and six females. All participants signed a consent form approved by Simon Fraser University. The signals were obtained using eight bipolar sensors, 16 channels, at a sampling rate of 1000 Hz, with an amplification gain of 500. Participants completed three different label sets; each had 16 hand gestures, a total of 48, shown on a screen with the object and gesture to perform. A further description of the gestures with images is in the original data collection study [29]. Signal samples are shown in Figure 2. With two sessions, 12 participants, three label sets, five repetitions, 12 features, and ten classifiers, we conducted more than 40,000 feature–classifier pairing experiments, which are described in Figure 3 and can be broken down as follows:

- **Sessions**: Two data collection sessions were performed. In the first, electrodes were on the forearm, whereas in the second, they were on the wrist, providing different scenarios to test if our hypotheses generalize to them.
- **Label groups**: For each session, three sets of gesture signals were collected. The first was 16 hand grasps of different objects taken from Cutkosky’s grasp taxonomy [30]. The second had 16 gestures from American Sign Language (ASL) [31]. The third contained hand positions [32], such as pronation and supination.
- **Repetitions**: Each gesture in each label group was performed by every participant 5 times, each lasting for 5 s. For details, see [29].

The dataset was chosen due to its variations to test hypotheses in multiple scenarios, such as different placements of the sensors.

### 2.2. Hypotheses’ Testing Experiments

To study the feature–model relationship, we evaluated models of differing assumptions, as we supposed that their assumptions were crucial to this relationship. We evaluated them on several sEMG features under a spectrum of feature window sizes and normalization ranges to test the feature–model compatibility hypothesis and find its optimal processing settings. We used the Python programming language for this study.

#### 2.2.1. Feature–Classifier Pairing Impact on Recognition Performance

We used various classifiers with different assumptions to thoroughly investigate the pairing impact. The list of classifiers used and their theoretical assumptions are:

- **Logistic regression (LR)** [33] assumes the linearity between data features and classes’ log probability.
- **Linear discriminant analysis (LDA)** [34] is another linear method that assumes each class’ data are Gaussian in the feature space with the same covariance matrix.
- **Support vector machine with linear kernel (SVM-LIN)** [35] is a linear classifier that depends on the fringe points of each of the classes to obtain the best linear boundary to separate the classes with allowed slack.
- **Quadratic discriminant analysis (QDA)** [36] is a quadratic model with similar assumptions to LDA, except for each class’s covariance matrix’s uniqueness.
- The **Naive Bayes (NB)** [37] model assumes the conditional independence between features given the data’s class.
- The **decision tree (DT)** [38] classifier sequentially splits the feature space based on learned thresholds.
- **K-nearest neighbors (KNN)** [39] stores the training data, without learning, to assign test data to the majority class of the K-nearest neighbors in the feature space.
- **Random forest (RF)** [40] is an ensemble of decision trees and is a critical classifier. In RF, a bagging technique, each tree is trained independently.
- **Gradient boosting (GB)** [41] is a boosting ensemble of decision trees, using misclassified points of a DT to enhance the training of the next one.
- **Support vector machine with radial basis kernel (SVM-RBF)** [35] has the same concept as SVM-LIN; however, it uses a kernel, a function resembling a metric in a hypothesized space, instead of the dot product.

Deep learning was excluded due to the dataset size. In each session, 400,000 samples were recorded per participant for each label group [29]. Applying our smallest window, 100 ms, yielded 4000 samples, which is very small for deep learning. In addition, a sizable amount of data is needed to ensure that even a small neural network is well-fit to the data and not underfitting. Otherwise, our results can be biased toward the collected data distribution and not the overall distribution. Another primary reason is the many hyperparameters require tuning for optimal performance. These hyperparameters require much more experiments on top of the 40,000 we conducted, rendering the study size too large to present adequately.

We paired the classifiers with twelve different commonly used sEMG features from the literature [24], each applied separately per sEMG electrode signal using a non-overlapping window. The features and their equations are included in Table 1.

#### 2.2.2. Influence of Window Size on Feature–Classifier Pairing

Nine windows sizes, 100, 250, 500, 750, 1000, 1250, 1500, 1750, and 2000 ms, with a non-overlapping stride, were included to monitor if some feature–classifier pairs surpassed others by altering the window size. This impact is essential for the performance–delay balance of gesture recognition. The pairs were then ranked by balanced accuracy. We regarded the processor computation time as negligible. Smith et al. [25] reported a time of 500 μs to compute their features, and processors have come a long way since then.

#### 2.2.3. Sufficiency of Signal Normalization Range

We evaluated four normalization ranges as a preprocessing step. The normalization parameters were obtained from training data only and used on each participant’s training and test data individually.

Signal normalization was applied as the first step before signal segmentation and filtering. Standard normalization was applied to the signals using the peak and lowest signal values for each sensor per subject, which was subject-specific.

Despite applying a single feature to the eight sEMG signals, we normalized the signals to compare the results between participants with varying signal amplitudes. The training data value can influence the classifiers. Normalization also ensured that such classifiers do not depend on specific sensors, whose signal amplitudes might change for the same participant, known as the inter-session variance for sEMG gesture recognition.

De Luca et al. [42] and Konrad et al. [43] explained that signal normalization for sEMG is essential to make sEMG independent of unwanted signal acquisition characteristics and to compare sEMG signals and their use between several users or variations in sensor placement, while our study included two different placements of the sensors among the 12 participants. We experimented with three ranges preserving the signal polarity: (−1, 1), (−2, 2), as well as normalization-free raw signals. We added (0, 1) normalization as a typical machine learning procedure. We analyzed these procedures using non-tunable feature–model pairs with 100, 500, and 1000 ms window sizes to remove any bias towards any single configuration.

### 2.3. Control Settings for Results’ Generalization

For the conclusions to be valid and extendable, we must guarantee that no information is passed from the testing data to the models. We carefully formulated conditions and rules to test any hypothesis in this study to hold this criterion.

#### 2.3.1. Conducting Trials on a Subset of Participants and Label Groups

We only used three random participants’ data to conduct the experiments. Using only 3 participants to dissect ensured that the study’s findings did not suffer from two hindrances. The first predicament arose from using data from one participant, which could incorporate too many anomalies; thus, the drawn conclusions cannot be generalized to the rest of the participants. Alternatively, selecting too many participants for analysis would jeopardize generalizing the findings because it would be prone to high bias.

Further, we used only the labels from the first session of the grasp gestures group to examine the research hypotheses without learning from all labels’ groups or sensor placements. The whole dataset was used to validate the research’s findings.

#### 2.3.2. Data Splitting by Repetition

We removed the first and last seconds of each 5 s gesture repetition to ensure the elimination of most noise in the data. The resulting 3 s did not include signals corresponding to the transition between gestures and belonged solely to the label gesture. We split each participant’s data by repetitions to ensure no data leakage between training and testing. In all experiments, we used four training repetitions (80%) and one test repetition (20%) with cross-validation, changing the repetitions assignment at each iteration.

For each feature, each participant’s data consisted of 8 columns, the features, except for the HIST feature, in which the number of columns was equal to the number of bins. After data cleaning and removing transitions between gestures, the rows were reduced from 400,000 to 240,000 for each participant per label set in each session. The number of rows varied depending on the window size used.

#### 2.3.3. Classifiers’ Training and Evaluation per Single Participant

For each participant in this study, we used 12 features, and per each feature, ten classification models were trained and evaluated using cross-validation. Each of the participant’s models was trained on their training subset and evaluated on their test subset for each cross-validation step without inter-participant testing, thus making them subject-specific. For each cross-validation fold, we removed a repetition from training to evaluate each hyperparameter configuration for the three subjects. We used the optimal hyperparameters to produce the cross-validation results on all the data. We used this cross-validation method to ensure that the reported accuracy results were not biased based on the testing repetition, but rather, the mean of multiple test repetitions’ performance.

We used the balanced accuracy metric as it is used for multi-class classification while addressing the class imbalance. The balanced accuracy metric weighs the accuracy of each class depending on the number of points of this class such that each class’s accuracy has the same contribution to the total accuracy. We interchanged the “balanced accuracy” and “accuracy” to mean the balanced accuracy metric throughout the paper.

#### 2.3.4. Hyperparameters’ Tuning for Optimizable Classifiers

We performed grid search cross-validation for six classifiers with the hyperparameters to optimize them. The grid is in Table 2.

We started by evaluating a hyperparameter value, then evaluated the effect of its increment or decrement. We iterated until increasing or decreasing beyond the previous two limits degraded the classifier’s performance. We then generated a range of values between the upper and lower limits.

We applied the grid search with cross-validation to each feature to yield optimized hyperparameters for each feature–model pair. The search ensured that the pair results were objective, as each model was tuned specifically for the feature used. Due to the number of hyperparameters investigated, we were unable to cover all of them, such as the kernel scale for SVM-RBF, for which we used the fixed value of $\frac{1}{n_{features}*VAR}$.

## 3. Results

### 3.1. Feature Normalization

**Figure 4:**
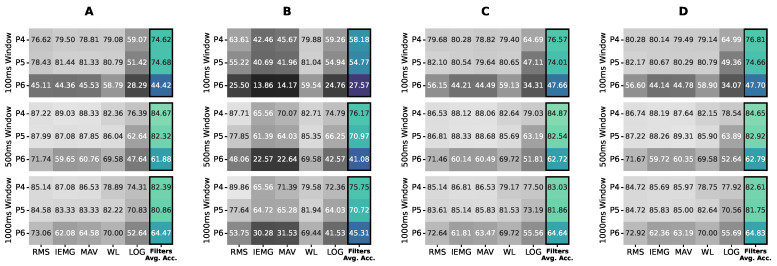
Averaged accuracies of logistic regression (LR), linear discriminant analysis (LDA), and quadratic discriminant analysis (QDA) for (**A**) no normalization, (**B**) (0, 1) normalization, (**C**) (−1, 1) normalization, and (**D**) (−2, 2) normalization on Participants 4, 5, and 6 data using non-tuned features with varying window sizes.

We tested the normalization first as a preprocessing step of the sEMG signals. In Figure 4, we applied four normalization ranges, each per subfigure, to the raw sEMG data, followed by applying the RMS, IEMG, MAV, WL, and LOG features, on the x-axis, with varying window sizes, as separate rows, to check if the effect was feature-independent without bias to a specific configuration. Figure 4 shows the averaged accuracy of non-tunable classifiers, LR, LDA, and QDA. As shown in Figure 4, (−1, 1), normalization gave the best results; thus, it was applied before processing the data for the investigation points to come.

### 3.2. Tunable Features and Classifiers’ Grid Search

**Figure 5:**
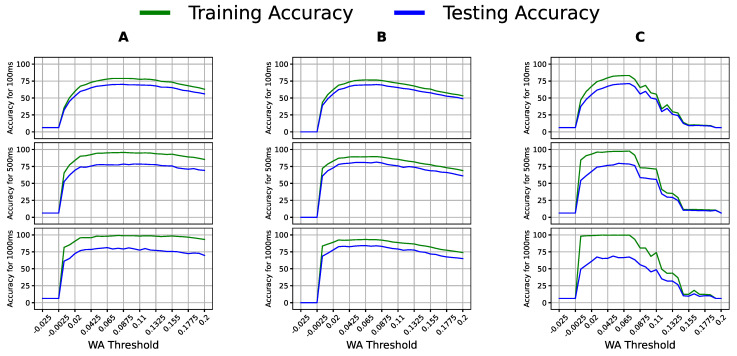
Classification accuracy of (**A**) logistic regression (LR), (**B**) linear discriminant analysis (LDA), and (**C**) quadratic discriminant analysis (QDA) with multiple WA threshold values on varying window sizes using the sEMG data of Participants 4, 5, and 6.

**Figure 6:**
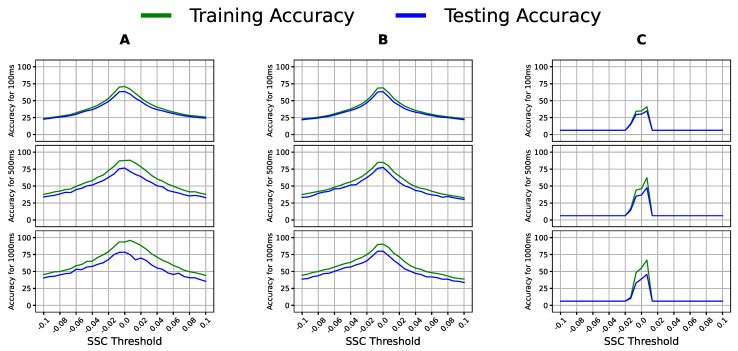
Classification accuracy of (**A**) logistic regression (LR), (**B**) linear discriminant analysis (LDA), and (**C**) quadratic discriminant analysis (QDA) with multiple SSC threshold values on varying window sizes using the sEMG data of Participants 4, 5, and 6.

We tuned the WA, SSC, and HIST features to optimize them first. In Figure 5 and Figure 6, showing one classifier per column and the window sizes as rows, the x-axis contains the threshold values, whereas the y-axis shows the accuracy.

Notably, Figure 5 shows a specific threshold range that gave the best accuracy across all classifier–window configurations; thus, the peak of this range was the optimal threshold for WA for this dataset. Similarly, SSC experienced a shared range of threshold values whose peak was chosen as the optimal threshold in Figure 6. The optimal values used for WA, SSC, and HIST were 0.065, 0.0066, and 30, respectively, and were used in the following experiments. We report the accuracy of two linear models, LR and LDA, and a quadratic model, QDA, to include the models’ different assumptions and varying complexities.

The grid search yielded the optimal hyperparameters for the models in Table 3 for each pair to tune each model to its paired feature. The grid results showed that the optimal hyperparameters were the same for all features and window sizes, indicating that these values tuned the models to the recognition task and not towards a specific setting, a feature, or a window size.

### 3.3. Processing Window Sizes

**Figure 7:**
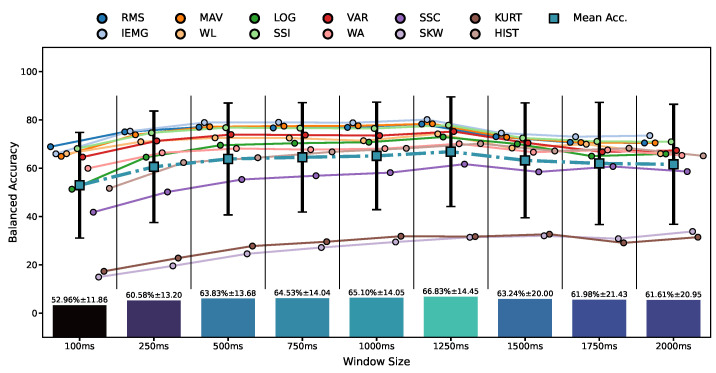
Features’ mean test accuracy for different window sizes of all classifiers using the sEMG data of Participants 4, 5, and 6.

From Figure 5 and Figure 6, we noticed that window expansion enhanced the accuracy notably, aligning with our hypothesis. To investigate if expanding the window was beneficial to the feature–classifier pairings, we illustrate the mean classification accuracy for the three participants, in Figure 7, per each model–feature pair with window sizes of 100, 250, 500, 750, 1000, 1250, 1500, 1750, and 2000. The optimal window size in Figure 7 is 1250 ms; thus, it was used in the experiments to come.

### 3.4. Feature–Classifier Pairs’ Compatibility and Ranking

**Figure 8:**
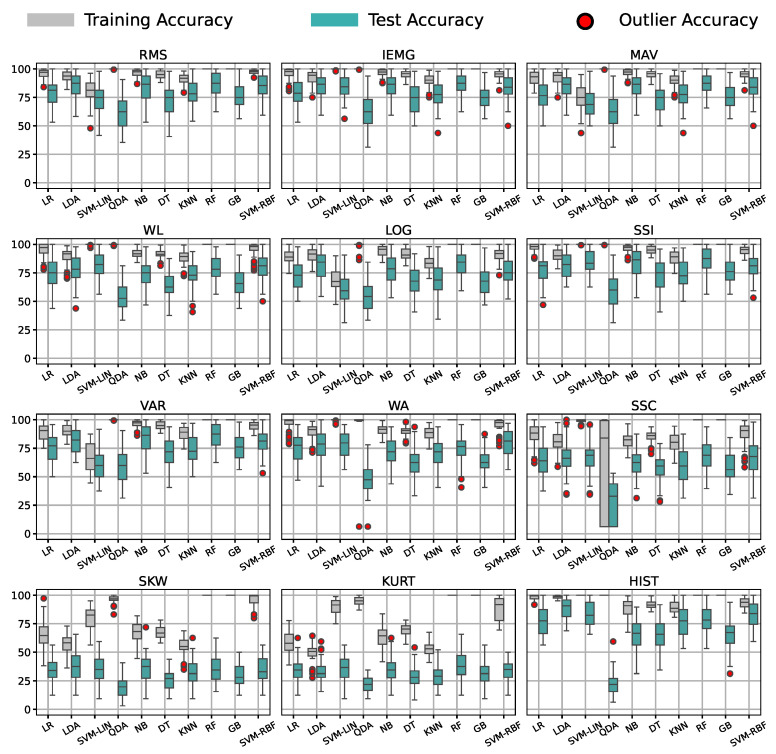
Distribution of accuracy for all feature–model pairs for the grasp label group on all participants’ data from the first session.

We finally evaluated the accuracy of all feature–classifier pairs, averaged over all participants’ data from the first session of the grasp labels using the optimal window size of 1250 ms. The distributions of the accuracy results are shown in Figure 8.

**Figure 9:**
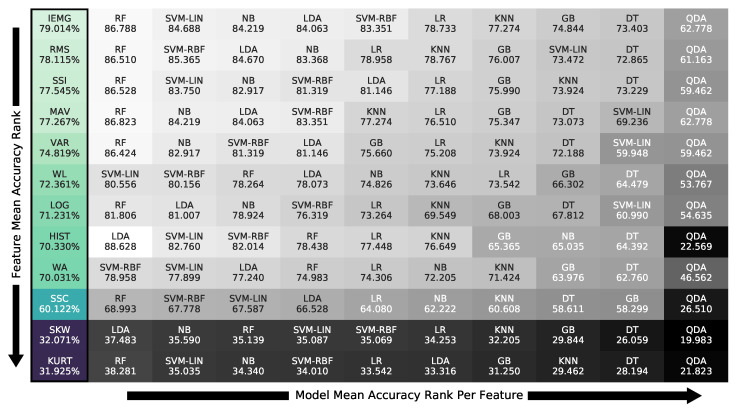
Feature accuracy ranking on the 1250 ms window with the classifiers’ sub-ranking using all participants’ grasp data.

Figure 8 is summarized in Figure 9, whose first column contains the descending order of the best features on the optimal window of 1250 ms from top to bottom. The models are ranked, in descending order, from left to right, for each feature, containing each feature–model pair’s accuracy. Figure 9 shows that the IEMG and RMS features, at 79.0% and 78.1% accuracy without threshold tuning, performed the best on average. At the same time, the SKW and KURT were much less efficient regardless of the classifier, averaging around 32.1% and 31.9%, respectively.

**Figure 10:**
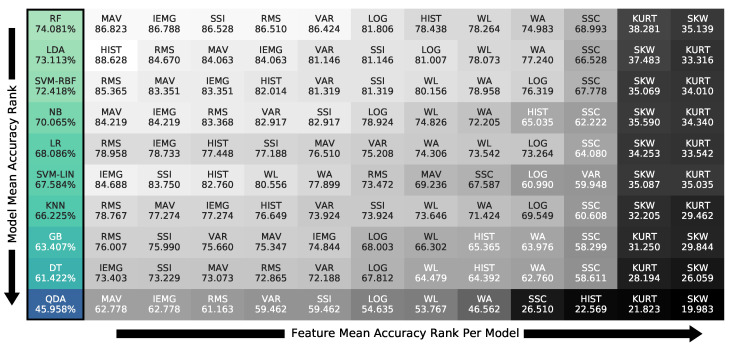
Classifier accuracy ranking on the 1250 ms window with the features’ sub-ranking using all participants’ grasp data.

The results in Figure 8 are also summarized in Figure 10 with the descending order of the models on the 1250 ms optimal window in the left-most column, from top to bottom. The features are ranked, in descending order, from left to right, for each model, showing the same pairs, but using the model as the primary index. The figure shows that the random forest model was the best for the grasp group with 74.1% accuracy averaged on all features, yet HIST-LDA was the top pair at 88.63%.

**Figure 11:**
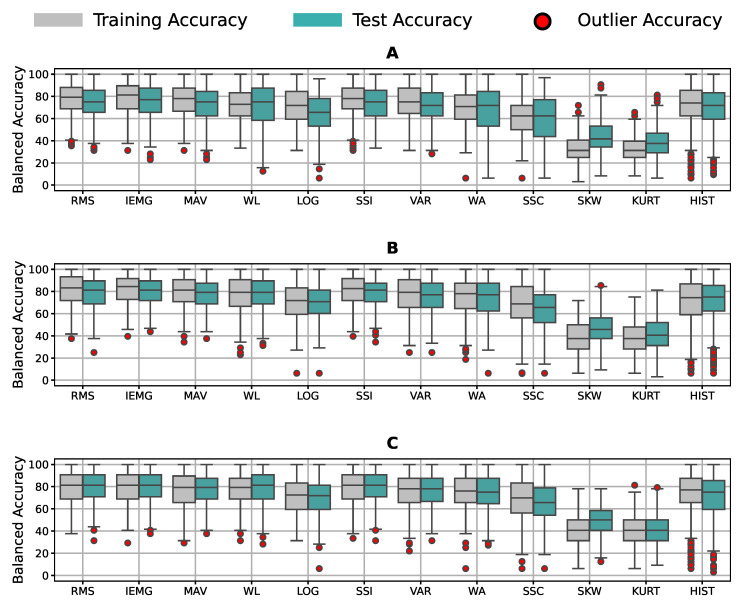
Accuracy distribution averaged over features for both sessions of (**A**) grasp gestures, (**B**) Sign Language gestures, and (**C**) unique movements gestures using all participants’ data.

Finally, we investigated if the results generalized for the Sign Language group, the hand movements group, and the other sensor placements. Figure 11 contains the mean accuracy of the features for participants’ signals from all sessions and sensor placements of all groups of labels. Most features gave the models a good performance, except with the SKW and KURT features. We noticed that the tunable features gave worse accuracy than others, except for the HIST feature; yet, it had the most outlier results.

## 4. Discussion

The feature–classifier ranking in Figure 9 supports the primary claim of the study of the prominence of their compatibility regardless of the models’ complexity, as models’ ranking changed per feature. This change is stressed by the SVM-LIN being the best model for the WL feature despite performing poorly on several features. We next found that the optimal accuracy of the 1250 ms window in Figure 7 proved the need to use relatively large window sizes to include sufficient information for optimal classification performance, as proposed. As for the normalization range, Figure 4 implies that the (−1, 1) range was sufficient for the best performance with no substantial enhancement by expanding the range.

Showing the need for proposed feature–model compatibility, we provide the ranking of the pairs in Figure 9 and Figure 10 to guide future research. The compatibility ranking showed simpler models, such as LDA, keeping up with ensemble models, such as RF, on certain features, such as HIST, RMS, and MAV, therefore giving accurate recognition with low computation; thus, it is necessary to choose the appropriate pair for sEMG gesture classification. For example, the ranking concluded the Willison amplitude (WL) feature as the best suited for linear models, as support vector machine with linear kernel (SVM-LIN) outperformed all, including ensembles. In opposition, SVM-LIN performed poorly on the VAR feature, while other linear ones surpassed the gradient boosting (GB) ensemble. We noticed the general behaviors of individual features or classifiers from their pairings’ performances.

Focusing on the models’ behavior in Figure 9, we note that random forest (RF) was the top classifier choice with a small variability compared to the rest, as noted in Figure 8. In contrast, quadratic discriminant analysis (QDA) performed worst on all feature pairings, indicating that the theoretical assumptions of QDA are not suitable for the classification task at hand. This claim was supported by the HIST feature’s high to acceptable results in Figure 9 on all classifiers, except for QDA. Such comparisons proved that the classifier’s theoretical assumptions, known as theoretical bias, and its compatibility with the feature play an immensely more significant role in the recognition process than its complexity, as the ensemble models surpassed the linear ones for some features, while failing for others.

Concentrating on the features’ results in the ranking, we concluded that IEMG, RMS, MAV, and SSI were the top-performing ones unconditionally, regardless of the window or the application type, real-time or offline recognition. Nonetheless, we realized that SKW, KURT, and SSC, mainly tunable features, offered a poor performance independent of the window in Figure 7 despite giving an acceptable performance for the three subjects’ data used for tuning. Such results showed that the tuned values did not generalize to the other participants. Figure 8 also concurs with this behavior, in which all models performed accurately on all pairings, but for the SSC, SKW, and KURT features, giving a low recognition accuracy all around with a distinctive overfitting issue. We inferred from these two notes that these features were signal-dependent in gesture recognition and must be tuned for each user individually, regardless of the classifier or feature window. Despite the HIST feature being one of the best-performing features, it yielded significant out-of-distribution results when generalized to other participants in Figure 11, confirming the need for user-specific feature tuning. We only report 12 commonly used features from the time domain due to the numerous features in the literature and the number of results discussed in the paper, from normalization ranges to pairing choice. However, more features such as wavelets or auto-regressive features can be explored in future work. Auto-regressive features depend on the auto-regression model’s order, requiring extensive investigation. Other features from the literature, such as time domain power spectral descriptors (TDPSDs), average intensity HOG (AIH), and time domain descriptors (TDDs) can also be considered for future research. Moreover, our research investigated single feature–classifier compatibility; yet, feature selection and combination’s effect on the compatibility could be a future research topic, using this study as a basis.

Concerning the feature window assumption, Figure 7 indicates that the 1250 ms window was optimal for all features. This supports our assumption that the optimal window for a classification task with this many labels, 16 gestures, is above the range of real-time applications, incorporating more information from signals for recognition. This result goes along with other findings in the literature [25]; however, the optimal window depends on the classification’s difficulty, reflected in the number of labels of 16 hand gestures. Thus, we analytically demonstrated that a small window does not encompass enough information to achieve the best performance. Furthermore, overextending the window in Figure 7 can have counter outcomes, allowing signal noise to deteriorate the recognition performance throughout differing control settings. However, a moderate window of 250 ms achieved acceptable accuracy for real-time applications, losing around 7% accuracy from the optimal window performance. This trade-off is a known accuracy–delay paradigm in the sEMG gesture recognition literature and investigated under specific environment controls [25]. However, our study verified it with various classifiers, features, and window sizes, thus having the slightest bias to any specific model or feature. Increasing the window size removes most disparities between the features, making the feature choice less impactful. The effect of the windows’ overlap and the window stride was not covered in this study, which could be analyzed in the future.

As for the signal normalization proposal, (−1, 1) normalization gave superior results for non-normalized features in Figure 4, whereas normalization between 0 and 1 gave a worse performance. From this observation, it is clear that the signals’ polarity plays a vital role in gesture recognition, as all procedures with negative to positive ranges performed better than 0 to 1 normalization. On top of that, equalizing the upper and lower limits, −1 to +1, performed better than non-normalized signals, yet expanding the range, −2 to +2, did not significantly impact the accuracy. Thus, a basic polarity-preserving signal normalization that balances the limits of the range is optimal regardless of the feature–classifier pair.

Experiments yielded very similar results for different sensor placements and other gestures, such as Sign Language and unique movements in Figure 11. Thus, our guidelines are generalizable to other recognition tasks and armband wearings.

Gesture classification treats false positive and false negative error equally; hence, the balanced accuracy metric is used throughout the literature in this area and our study. Despite that, statistical analysis and other metrics might give an insight into the feature–classifier relationship as a future point, which was excluded due to the number of points investigated, for example, statistic techniques such as Shapley additive explanations (SHAP) or maximum relevance minimum redundancy (MRMR).

Other aspects of signal variation can be future research points for the feature–classifier compatibility. The sampling rate of the signals and noise elimination applied as preprocessing are future investigation areas to conclude about their effect on the proposed compatibility. Validating the findings of this study on more participants’ data who differ in ethnicity and dominant hand is a future point to research. Moreover, there are other hyperparameters of the classifiers we used to include. However, due to the study’s size, we could not add all of them. The inclusion of more hyperparameters of the classifiers will further solidify the results of the study in the future, such as SVM scale optimization for the RBF kernel exclusion.

## 5. Conclusions

This paper introduced an unexplored aspect of surface electromyography (sEMG) gesture recognition, which we labeled feature–classifier compatibility. We showed that, under varying signal preprocessing choices, hyperparameter tuning, window sizes, and for different users, a relationship exists between the pairing choice of the classifier and EMG feature. This conclusion was supported by the results of the experiments with more than 40,000 feature–classifier pairs. We concluded that pairing compatibility was the most influential for sEMG gesture recognition, even more than the quality of the classifier or feature used. Our research avoided bias to a particular feature or classifier, as most studies focus on an application that uses only one classifier or feature. The pairing compatibility is ranked as a foundation for future research as it gave the best pairing with a pre-chosen feature or classifier in an application or a feature–classifier pair with comparable performance. Moreover, more studies are needed on this point, such as extending the compatibility investigation to more features or feature selection.

In addition, we presented the best normalization, window size, and hyperparameter tuning we obtained through out our experiments. These advantages are beneficial to any gesture recognition application, specifically those with limited computational capabilities.

The compatibility’s importance was evidenced by the SVM-LIN achieving the best on the WL feature, 80.56% accuracy, and the second-worst on the MAV feature, 69.24% accuracy. The ranking showed that linear models compete with complex ones on specific features, as the HIST-LDA feature–model pair performed best with 88.63% accuracy for 16 gestures without any further optimization. To highlight the importance of the suggested compatibility, Mendes et al. [24] obtained 94% accuracy for six gestures only after dimensionality reduction and feature selection. We suggest feature selection and dimensionality reduction as future optimization research to enhance our compatibility ranking performance further. Thus, it enables low-computation gesture recognition and implies that the models’ complexity in gesture recognition is not as effective as believed. We presented the optimal hyperparameters under different scenarios for future research of various conditions.

Secondly, we found that the 1250 ms window was optimal for this recognition task’s 16 hand gestures. In comparison, raising or lowering its size reduced the classification accuracy. Nonetheless, real-time applications that require window sizes of less than 300 ms would benefit from the fact that the 250 ms window provided acceptable accuracy with a 7% reduction from optimal performance. We further concluded that the inequalities between feature performances faded for larger window sizes.

As for the normalization, the common signal normalization, from −1 to +1, was unconditionally most suited for gesture recognition independent of the task. Other ranges were equally or less valuable, particularly ones removing the signal polarity.

Other factors can be investigated in future studies regarding the proposed compatibility, such as the inclusion of more participants, features, and other metrics for analysis.

## Figures and Tables

**Table 1:** Signal features’ equations and variables.

Feature	Formula	Variables
Root mean square **(RMS) [19]**	(1) $$RMS=\sqrt{\frac{1}{N}\sum_{i=1}^{N}x_{i}^{2}}$$	*N*: window size $x_{i}$: current reading
Integrated EMG **(IEMG) [19]**	(2) $$IEMG=\sum_{i=1}^{N}\left|x_{i}\right|$$	*N*: window size $x_{i}$: current reading
Mean absolute value **(MAV) [19]**	(3) $$MAV=\frac{1}{N}\sum_{i=1}^{N}\left|x_{i}\right|$$	*N*: window size $x_{i}$: current reading
Waveform length **(WL) [19]**	(4) $$WL=\sum_{i+1}^{N-1}\left|x_{i+1}-x_{i}\right|$$	*N*: window size $x_{i}$: current reading $x_{i+1}$: next reading
Log detector **(LOG) [19]**	(5) $$LOG=\exp\left(\frac{1}{N}\sum_{i=1}^{N}\log(|x_{i}\left|\right)\right)$$	*N*: window size $x_{i}$: current reading
Simple square integral **(SSI) [19]**	(6) $$SSI=\sum_{i=1}^{N}x_{i}^{2}$$	*N*: window size $x_{i}$: current reading
Variance of EMG **(VAR) [19]**	(7) $$VAR=\frac{1}{N-1}\sum_{i=1}^{N}{\left(x_{i}-\bar{x}\right)}^{2}$$	*N*: window size $x_{i}$: current reading $\bar{x}$: signals’ mean
Willison amplitude **(WA) [19]**	(8) $$\begin{array}{c} WA=\sum_{i=1}^{N-1}f(|x_{i}-x_{i+1}\left|\right)\\ where\;f\left(x\right)=\left\{\begin{array}{c} 1,\;if\;x\geq \delta \\ 0,\;\mathrm{otherwise} \end{array}\right. \end{array}$$	*N*: window size $x_{i}$: current reading $x_{i+1}$: next reading δ: threshold value
Slope sign change **(SSC) [19]**	(9) $$\begin{array}{c} SSC=\sum_{i=2}^{N-1}f\left(\left[x_{i}-x_{i-1}\right]\times \left[x_{i}-x_{i+1}\right]\right)\\ where\;f\left(x\right)=\left\{\begin{array}{c} 1,\;if\;x\geq \delta \\ 0,\;\mathrm{otherwise} \end{array}\right. \end{array}$$	*N*: window size $x_{i-1}$: prior reading $x_{i}$: current reading $x_{i+1}$: next reading δ: threshold value
Skewness **(SKW)**	(10) $$SKW=\frac{\sum_{i=1}^{N}{\left(x_{i}-\bar{x}\right)}^{3}/N}{\delta^{3}}$$	*N*: window size $x_{i}$: current reading $\bar{x}$: signals’ mean δ: signals’ std. dev.
Kurtosis **(KURT)**	(11) $$KURT=\frac{\sum_{i=1}^{N}{\left(x_{i}-\bar{x}\right)}^{4}/N}{\delta^{4}}$$	*N*: window size $x_{i}$: current reading $\bar{x}$: signals’ mean δ: signals’ std. dev.
Signal histogram **(HIST)**	(12) $$HIST=\frac{1}{S}\sum_{j=1}^{S}\sum_{i=1}^{N}I\left(B_{l}<x_{j,i}\leq B_{h}\right)$$	*N*: window size $x_{j,i}$: current reading *j*: sensor number $B_{l}$, $B_{h}$: bin bounds

**Table 2:** Grid search values for classifiers’ hyperparameters.

Classifier	Hyperparameter	Grid Values
SVM-LIN	C	0.1, 1, 5, 25, 45, 65, 85, 105, 125, 145
DT	Pruning Coeff Split Min Samples	0.0, 0.01, 0.02, 0.03, 0.04, 0.05 5, 10, 15
KNN	Distance Metric Neighbors Weights Neighbors (K)	Minkowski, Euclidean Uniform, Distance 5, 10, 15
RF	Pruning Coeff. # Base Models	0.0, 0.01, 0.02, 0.03, 0.04, 0.05 25, 50
GB	Pruning Coeff. # Base Models	0.0, 0.0025, 0.005, 0.0075, 0.01, 0.0125 25, 50
SVM-RBF	C	0.1, 1, 10, 20, 30, 40, 50, 60, 70, 80, 90

**Table 3:** Grid search optimal hyperparameters for classification models using the sEMG data of Participants 4, 5, and 6.

Classifier	Hyperparameters
SVM-LIN	C = 80 (for Win = 100), 85 (for other Win Sizes)
DT	Pruning Coeff = 0; Split Min Samples = 5
KNN	Distance Metric = Minkowski Neighbors Weights = Uniform; Neighbors (K) = 5
RF	Pruning Coeff = 0; # Base Models = 50
GB	Pruning Coeff = 0; # Base Models = 50
SVM-RBF	C = 90

## Data Availability

Restrictions apply to the availability of the dataset used in the study. The dataset was obtained from the previous study “Jiang, Xianta & Merhi, Lukas-Karim & Xiao, Zhen & Menon, Carlo. (2017). Exploration of Force Myography and surface Electromyography in Hand Gesture Classification. Medical Engineering & Physics. 41. In Press. 10.1016/j.medengphy.2017.01.015.” [29] with the consent of the Data Custodian and the Ethics Board of Simon Fraser University.
